# Efficacy and Safety of Apixaban versus Dalteparin as a Treatment for Cancer-Associated Venous Thromboembolism: A Systematic Review and Meta-Analysis

**DOI:** 10.3390/medicina59101867

**Published:** 2023-10-20

**Authors:** Miguel A. Arce-Huamani, Joshuan J. Barboza, José Fabián Martínez-Herrera, J. Smith Torres-Roman, Jorge L. Maguiña

**Affiliations:** 1 Facultad de Ciencias de la Salud, Universidad Cientifica del Sur, Lima 15067, Peru; jorge.luis.maguina@gmail.com; 2 Cancer Research Networking, Universidad Cientifica del Sur, Lima 15067, Peru; jfabianmd@gmail.com (J.F.M.-H.); jstorresroman@gmail.com (J.S.T.-R.); 3 Centro de Investigación en Epidemiología y Medicina Basada en Evidencia, Universidad Norbert Wiener, Lima 13007, Peru; jbarbozameca@gmail.com; 4 Cancer Center, Medical Center American British Cowdray, Mexico City 01120, Mexico; 5 Latin American Network for Cancer Research (LAN–CANCER), Lima 11702, Peru

**Keywords:** apixaban, dalteparin, meta-analysis, venous thromboembolism, cancer

## Abstract

*Background and Objectives*: Venous thromboembolism (VTE) is common in cancer patients. Anticoagulant therapy with low-molecular-weight heparins (LMWHs) and direct oral anticoagulants (DOACs), such as dalteparin and apixaban, have demonstrated efficacy and safety. However, more comparative research on these drugs is still needed. This study aimed to synthesize evidence on the efficacy of apixaban compared to dalteparin in reducing recurrent VTE, major bleeding, and clinically relevant non-major bleeding associated with cancer. *Materials and Methods*: We systematically searched the PubMed, Scopus, Web of Science, Embase, Cochrane Library, and ClinicalTrials databases up to 5 January 2023 for randomized controlled trials comparing apixaban versus dalteparin as a treatment for cancer-associated VTE. Five studies were included. Effects according to meta-analyses were reported as relative risks (RRs) and their 95% confidence intervals (CIs). *Results*: It was found that 33 of 734 (4.5%) patients treated with apixaban and 56 of 767 (7.3%) with dalteparin had recurrent VTE as an efficacy outcome (RR 0.49, 95% CI 0.15–1.58, I^2^ 38%). Major bleeding occurred in 25 of 734 patients treated with apixaban (3.4%) and 27 of 767 patients treated with dalteparin (3.5%) (RR 1.29, 95% CI 0.31–5.27, I^2^ 59%). Likewise, clinically relevant non-major bleeding occurred in 64 of 734 patients treated with apixaban (8.7%) and 46 of 767 (5.9%) patients treated with dalteparin (RR 1.52, 95% CI 1.05–2.19, I^2^ 0%). *Conclusions*: Apixaban showed a lower risk of recurrent VTE than dalteparin in patients with cancer-associated VTE, albeit with no statistical difference. Statistical significance was observed for no major clinically relevant bleeding but not for major bleeding.

## 1. Introduction

Venous thromboembolism (VTE) is the third most important vascular disease in the general population. Cancer patients are 4 to 7 times more likely to develop VTE, with it being the second cause of death among these patients [1,2]. The incidence of recurrent VTE is 9.6%, reaching 22.1% in the first 6 months after the initial diagnosis of cancer [3]. Anticoagulant therapy is recommended to prevent VTE in high-risk patients (KHORANA score >2) and to treat those who have already had a thrombotic event [4]. However, this therapy carries a significant risk of bleeding, the most serious adverse reaction. Low-molecular-weight heparins (LMWHs) and direct oral anticoagulants (DOACs) are the most widely used anticoagulant treatments [3,5]. Dalteparin, an LMWH, is recommended in patients with cancer-related VTE, as it has been associated with a lower incidence of osteoporosis and heparin-induced thrombocytopenia [6]. Among DOACs, apixaban has been shown to have a lower risk of systemic cerebrovascular disease/embolism and major bleeding [7]. However, the cost of the aforementioned drugs is approximately USD 64 and USD 106 for apixaban and dalteparin, respectively, making access by the general public difficult [8,9]. Therefore, it is necessary to propose strategies for their acquisition.

Various studies have addressed DOACs and LMWHs as treatment for cancer-associated VTE [10,11], with new drugs such as apixaban and dalteparin having demonstrated efficacy and safety compared to other common treatments [12,13]. Regarding thromboprophylaxis, the efficacy and safety of apixaban have been reported in a randomized clinical trial (RCT) with 130 patients with gastrointestinal cancer, in whom it was shown to reduce the incidence of thromboembolism to 4.6% compared to 20% in the placebo group [14]. In another RCT, in 365 patients stratified according to the presence or absence of metastatic cancer, thromboprophylaxis with apixaban was associated with a significantly lower rate of VTE, with a hazard ratio of 0.55 and 0.34, respectively, compared to the placebo [15]. For its part, in an RCT in patients with VTE associated with gynecological cancer, dalteparin showed a lower occurrence of major bleeding in 5% compared to 7.8% in those who received rivaroxaban as treatment [16]. However, no study has synthesized the results of treatment with apixaban and dalteparin for VTE in cancer patients, which is important information.

Consequently, the study authors note that evaluation of the efficacy and safety of the treatment of VTE associated with cancer remains challenging due to the various studies available that expand the treatment options. Likewise, the adverse reactions of these treatments can be harmful for patients, making it important to synthesize the evidence between them. Apixaban and dalteparin have undergone several studies demonstrating their capacity to reduce major bleeding and the recurrence of VTE in cancer patients. However, despite the number of RCTs comparing these drugs with other LMWHs and DOACs, no study has compared the efficacy and safety of apixaban and dalteparin.

Therefore, the purpose of our study was to synthesize evidence on the efficacy and safety of apixaban versus dalteparin in reducing recurrent VTE, major bleeding, and clinically relevant non-major bleeding associated with cancer.

## 2. Materials and Methods

We used the PRISMA report for systematic reviews, and meta-analyses [17] for writing our systematic review. The version of our protocol was registered in the International Prospective Register of Systematic Review (PROSPERO) with the code CRD42021275583.

### 2.1. Data Sources and Searches

From inception to 5 January 2023, we searched the following databases: PubMed, Scopus, Web of Science, Embase, and Cochrane Central Register of Controlled Trials (CENTRAL) for studies evaluating the efficacy of apixaban versus dalteparin as a treatment for cancer-associated VTE, and conducted a search of ClinicalTrials to identify ongoing or completed RCTs related to our review. The decision was made to include dalteparin as the sole LMWH (control group) due to the lack of studies that exclusively compared these two drugs, both of which have shown promising results in cancer-associated VTE. This choice aims to maintain consistency within the comparison and reduce result variability by standardizing the comparison, thus avoiding differences between different low-molecular-weight heparins.

In the search strategy, we used the terms “Apixaban”, “Dalteparin” and “Venous thromboembolism” and their MESH synonyms using the Peer Review of Electronic Search Strategies Checklist [18]. Our team developed the search strategy in PubMed, and it was adapted to the different bibliographic databases that were mentioned (Supporting Information). No language restrictions were applied.

### 2.2. Selection of Studies and Data Extraction

Studies that met the following criteria were included: (1) experimental study RCT, phase 2 or 3, parallel or crossover; (2) studies that included patients with cancer of any etiology and presenting with VTE; (3) treatment with apixaban as experimental/intervention group with a duration of 6 months; and (4) dalteparin as a control group with a duration of 6 months of treatment. Likewise, studies conducted in animals, duplicates, conference abstracts, case reports and series, observational studies, review articles, systematic reviews, and editorials or comments were excluded. The outcomes were classified in terms of efficacy and safety, which are used in RCTs; the efficacy outcomes were prevention of venous thrombosis and the safety outcomes were bleeding [19]. In our study, the efficacy outcome was recurrent VTE, which was defined as thrombosis of a venous site that was not previously affected, or a previous history of resolution interval of the incident thrombus [20]. On the other hand, the safety outcomes were major bleeding, defined as obvious bleeding accompanied by a decrease in hemoglobin of ≥2 g/dL; or transfusion of ≥2 red cell packet units; or intracranial, intraspinal/epidural, intraocular, retroperitoneal, pericardial, intraarticular, intramuscular hemorrhage with compartment syndrome or fatal [21]; and clinically relevant non-major bleeding was defined as overt bleeding that does not meet the definition of major bleeding but is associated with medical intervention, unscheduled medical attention, or temporary discontinuation of anticoagulant therapy [22].

Two reviewers (MAAH and JJB) independently screened the titles and abstracts of the selected articles to choose potentially relevant articles. After finding potential studies to be included, four authors (MAH, JJB, JSTR, and JFMH) independently read the full text of each selected article. If an article did not meet one or more selection criteria, it was excluded from the study. Discrepancies were resolved by consensus among the team of researchers at each stage. We used Rayyan QCRI software Version 1.2.1 (Qatar Computing Research Institute, Doha, Qatar) to carry out the study selection process [23]. Finally, two authors (MAH and JJB) extracted data from the studies using a standardized data extraction sheet performed in Microsoft Excel. The following information was extracted: author, year, country, study design, number of participants, intervention, comparator, duration of treatment, efficacy and safety outcomes, mean age, percentage male, active cancer, metastatic cancer, ECOG status greater than or equal to 2, solid tumor, hematological malignancy, body weight—median (standard deviation), creatinine clearance 30–50 mL/min, platelet count 50–100,000/mm^3^ and incidental pulmonary embolism at the time of diagnosis.

### 2.3. Assessment of the Certainty of the Study Evidence and Risk of Bias of the Studies

The certainty of the evidence was assessed using the GRADE methodology [24], covering all five aspects: risk of bias, inconsistency, indirect evidence, imprecision, and publication bias. The certainty of the evidence was assessed by the outcome, and we used the GRADEpro GDT software version 2021 because it allows for summarizing and presenting information for healthcare decision making by creating summary of findings (SoF) tables.

We used the 2019 Risk of Bias (RoB) 2.0 tool to assess the risk of bias of the RCTs included in this study [25]. This tool assessed several domains from which bias may arise: Risk of bias by domain follows an algorithm to conclude low risk, some concerns, or high risk per domain and per trial. The evaluation of the RoB 2.0 was carried out independently by two authors (MAH and JJB), and discrepancies were resolved by discussion or the consultation of a third author (JMQ).

### 2.4. Data Synthesis and Analysis

Statistical analyses were performed with Review Manager 5.3 (RevMan 5.3) (The Cochrane Collaboration, Copenhagen, Denmark). An inverse-variance random effects meta-analysis evaluating the effect of apixaban versus dalteparin on outcomes was performed. The treatment effects were reported as relative risks (RRs) and their 95% confidence intervals (CIs). CIs for effects were adjusted using the Hartung–Knapp method, and between-study tau2 variance was calculated using the Paule–Mandel method. The heterogeneity of the effects between the studies was quantified with the I2 statistic (an I2 > 60% corresponds to moderate heterogeneity) [26].

We performed an outcomes analysis according to the following criteria: apixaban dose (10 mg dose twice daily for the first 7 days, followed by 5 mg twice daily) and dalteparin dose (200 IU dose per kilogram of body weight once daily for the first month, followed by 150 IU per kilogram once daily) and recurrence of VTE, major bleeding, and no major clinically relevant bleeding; the interaction test was used where the *p*-value < 0.05 indicates an effect modification by outcomes [27]. Review Manager 5.3 (RevMan 5.3) software (The Cochrane Collaboration, Copenhagen, Denmark) was used for the meta-analysis.

## 3. Results

### 3.1. Subsection

#### 3.1.1. Selection of Studies 

**Figure 1:**
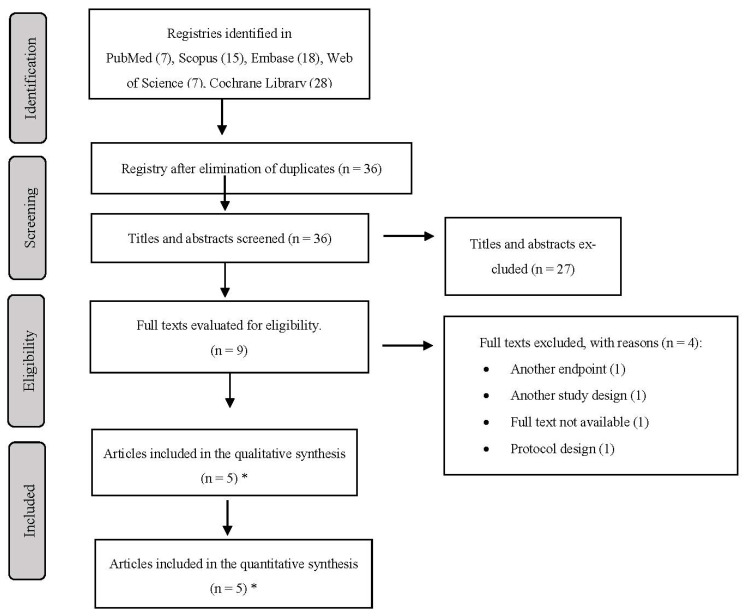
PRISMA 2009 flow chart. * 5 articles: 3 on the Caravaggi study, 1 on the ADAM study and 1 on the PRIORITY study.

The flowchart summarizing the study selection process is shown in Figure 1. In the initial search, we found a total of 75 records. After excluding duplicate studies, 36 studies were retained. Subsequently, during the evaluation of titles and abstracts, 27 more records were excluded. Finally, during the full-text evaluation, four articles were excluded due to having another outcome, another study design, not being available in full text, or due to the protocol design. Finally, three studies published in the five articles selected for the synthesis of the information, with three corresponding to the Caravaggio study [28,29,30], one study corresponding to the ADAM TVE trial (VTE) [31], and one phase II study corresponding to the PRIORITY trial [32].

#### 3.1.2. Description and Characteristics of the Studies

The characteristics of the studies included are presented in Table 1. For this systematic review, three studies were included; these were open label RCTs. The efficacy outcome (recurrent VTE) and safety outcomes (major bleeding and clinically relevant non-major bleeding) were reported by all three studies. The doses in the intervention and comparator were the same in the three clinical trials (Caravaggio, ADAM, and PRIORITY) with a duration of 6 months of treatment. The mean and median age ranged from 64 to 67 years, the male-to-female ratio was similar, and more than 60% of the patients had metastatic cancer.

#### 3.1.3. Assessment of the Certainty of the Study Evidence and Risk of Bias of the Studies

**Figure 2:**
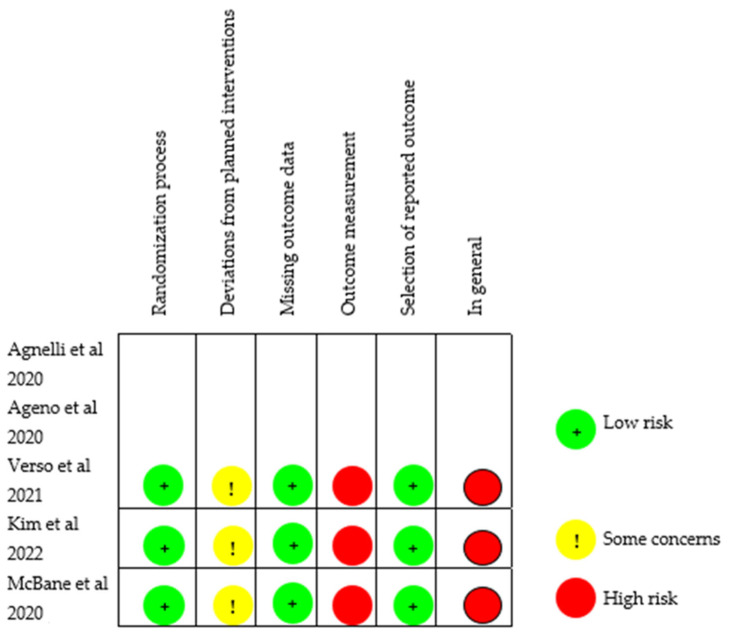
2019 Cochrane risk of bias (RoB) Tool 2.0 [25,28,29,30,31,32].

For the risk of bias, the Cochrane RoB 2.0 tool presented in Figure 2 was used. All three trials had some concerns in one dimension (deviations from planned interventions) and high risk in another (outcome measurement), and thus, overall, all three studies had a high risk of bias. The high risk of bias rating in outcome measurement and deviations from planned intervention was based on several factors. This includes insufficient blinding, for both participants and researchers, which can allow for unconscious influence on the results. Deviations from the planned intervention, such as treatment non-compliance or unforeseen changes, can bias the results. Furthermore, inadequate follow-up can contribute to an increased risk of bias in the research.

We used the GRADEpro GDT software version 2021 to create the summary table of the outcomes shown in Table 2. Three outcomes (one of efficacy and two of safety) were evaluated. The degree of certainty of the evidence was very low in these studies since the participants knew which drug they were receiving by the route of administration, and therefore, the blinding was lost. The relative effect with a 95% CI was not statistically significant for two outcomes (recurrent VTE and major bleeding).

#### 3.1.4. Outcomes Analysis 

**Figure 3:**
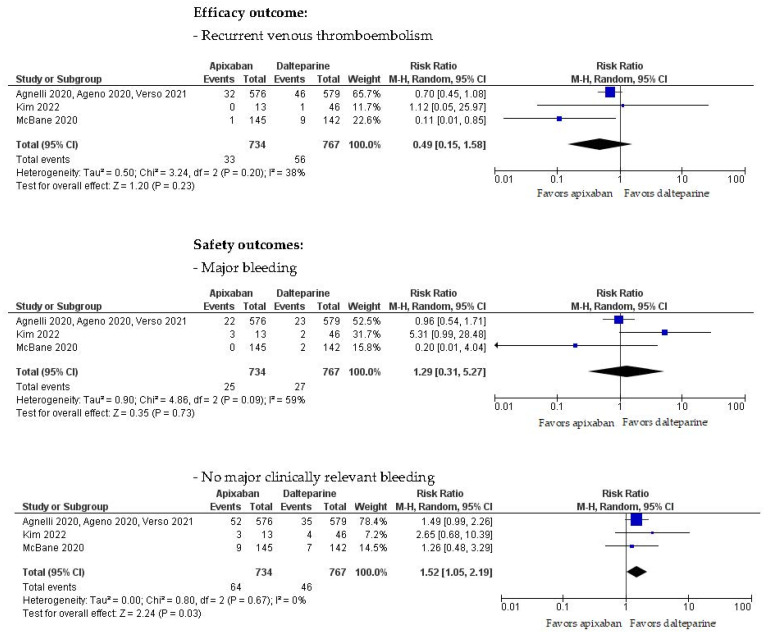
Forest plot of studies comparing apixaban and dalteparin [26,28,29,30,31,32].

The outcome analysis was performed according to the efficacy (primary) and safety (secondary) outcomes using the Forest plot presented in Figure 3. Thirty-three of the 734 (4.5%) patients treated with apixaban and 56 of 767 (7.3%) receiving dalteparin had recurrent VTE as the efficacy outcome (RR 0.49, 95% CI 0.15–1.58, I^2^ 38%). Major bleeding occurred in 25/734 patients treated with apixaban (3.4%) and 27/767 with dalteparin (3.5%) (RR 1.29, 95% CI 0.31–5.27, I^2^ 59%). Likewise, clinically relevant non-major bleeding occurred in 64/734 patients treated with apixaban (8.7%) and 46/767 (5.9%) with dalteparin (RR 1.52, 95% CI 1.05–2.19, I^2^ 0%).

## 4. Discussion

According to the results of our systematic review, apixaban and dalteparin did not significantly reduce the RR of recurrent VTE and major bleeding in patients with cancer-associated VTE. However, dalteparin safely reduced the RR of clinically relevant non-major bleeding with anticoagulant therapy compared to apixaban. Analysis by outcomes due to recurrent VTE was the main finding, and clinically major bleeding and non-major bleeding were the secondary findings of the present study. The risk of bias was high in the three studies evaluated, while the degree of certainty of the evidence was very low in all three studies.

Various guidelines have established DOACs and LMWHs as an alternative to usual treatment to counteract side effects, such as bleeding and thrombocytopenia, in the prophylaxis and treatment of vascular diseases in cancer patients [33,34], with DOACs being more accepted due to their oral administration [35,36]. Multiple meta-analyses comparing LMWH and DOAC treatments were performed [37,38,39,40,41,42,43]. Some outcomes evaluated in each treatment showed no significant differences between LMWH and DOAC, with the latter being used as another treatment option for cancer-associated VTE [37,38,39,40,41,42,43]. Other studies have concluded that among the drugs used for the prevention and treatment of VTE in cancer patients, DOACs have a greater efficacy and a relatively low risk of bleeding, and apixaban is the most effective and has the lowest risk of bleeding [37,38,39,40,41,42,43,44]. However, some studies have reported that DOACs have an increased risk of major bleeding in patients with gastrointestinal and genitourinary malignancies [37,38,39,40,41,42,43].

A meta-analysis of four RCTs concluded that DOACs are more effective in the treatment of VTE associated with malignancy compared to LMWH, although an increased risk of clinically relevant combined major or non-major bleeding was observed [45]. Another meta-analysis of eight RCTs comparing DOACs with LMWH and vitamin K antagonists (VKAs) concluded that DOACs are the optimal treatment for cancer-associated VTE, have a similar or slightly increased risk of bleeding compared to LMWH, are a safer alternative to VKAs, and have a promising lowering effect on mortality, regardless of cancer status in these patients [46]. The recently published phase II PRIORITY RCT involving 90 patients with active cancer reported that DOAC therapy further increased the risk of bleeding compared to dalteparin in patients with active advanced upper gastrointestinal, hepatobiliary, or pancreatic cancer. Therefore, great care is needed when selecting anticoagulant therapy for cancer-associated VTE in high-risk patients [32]. Two meta-analyses had similar conclusions to those of our study, describing that LMWH drugs had fewer bleeding episodes compared to DOACs [47,48]. However, our results included two additional components, an assessment of the quality and certainty of the evidence, and an outcomes analysis of efficacy and safety according to the type of bleeding and in patients with cancer.

In our analysis using the GRADE summary results table, we found that patients receiving apixaban presented statistically significant, clinically relevant non-major bleeding, with an anticipated absolute effect greater than 91 per 1000 compared to those receiving dalteparin with 60 per 1000 (RR 1.52; 95% CI, 1.05 to 2.19). On the other hand, there was no statistical difference in major bleeding for patients using both drugs. However, recurrent VTE was non-significantly lower in patients with apixaban compared to dalteparin. The number of participants in the RCTs evaluated add up to 1501, which is a considerable number for the synthesis of evidence for the two drugs. However, based on the very-low-certainty evidence, the effect of the drugs is uncertain; therefore, the use of apixaban cannot be recommended because it may increase the risk of clinically relevant non-major bleeding compared to dalteparin.

In the various meta-analyses evaluating DOACs versus LMWH described above for the treatment of cancer-associated VTE, the evaluation was carried out jointly and not directly between each drug. Therefore, in our study, we chose the most effective and safest DOACs and LMWH drugs, apixaban and dalteparin, with the results showing the efficacy and safety of these drugs in the different types of bleeding and encouraging the need for more RCTs and a subsequent meta-analysis of these trials.

The various factors that contribute to the risk of bleeding in cancer patients are a challenge for physicians administering anticoagulation treatment in this population. These factors involve the pathophysiology of cancer, thrombocytopenia, chemotherapy treatment, and subsequent renal failure, which generate hemostatic instability. Taking all these factors into account the evidence as to whether LMWH and DOACs have a similar efficacy and safety remains insufficient and thus, more RCTs on the use of these drugs is necessary to achieve more solid and significant conclusions.

Our study has some limitations that must be considered. The small number of studies available comparing apixaban and dalteparin did not allow for a greater synthesis of the results; however, the number of participants in the studies evaluated provide the relative risks with respect to each outcome. A strength of this study is the methodology used, describing the certainty of the study evidence and risk of bias of the studies included in a systematic review and meta-analysis. Another strength is that the PRIORITY study was added, for the first time, to a meta-analysis for the summary of its results. Furthermore, it is important to mention that it is not the time to contemplate the inclusion of these medications in the national cancer plan when the evidence of their efficacy and safety is still unclear.

## 5. Conclusions

Patients with cancer-associated VTE treated with apixaban showed a lower risk of recurrent VTE (safety outcome) compared to dalteparin, albeit with no statistical difference. In the evaluation of clinically relevant non-major bleeding, there were statistical differences between the two drugs. Likewise, the certainty of the evidence of the studies was very low and the risk of bias was high; thus, it is still not possible to suggest that one of the evaluated treatments is more effective and safer than the other.

## Figures and Tables

**Table 1:** Characteristics of the studies included.

Author, Year	Study Design	Number of Participants	Intervention and Comparator	Dose	Treatment Duration	Efficacy and Safety Outcomes	Average Age	Male Gender	Metastatic Cancer	Creatinine Clearance 30–50 mL/min	Platelet Count 50–100,000/mm^3^
Agnelli et al., 2020 [28]; Ageno et al., 2020 [29]; Verso et al., 2021 [30]	Randomized and open trial	1155	Apixaban	Apixaban 10 mg orally twice daily for the first 7 days and 5 mg twice daily.	6 months	Efficacy outcome: - recurrent VTE Safety outcome: - Major bleeding - No major clinically relevant bleeding	67.2	50.7%	67.5%	8.9%	3.6%
			Dalteparin	Subcutaneous dalteparin (200 IU/kg for 1 month followed by 150 IU/kg once daily).			67.2	47.7%	68.4%	10.5%	3.8%
Kim et al., 2022 [32]	Multicenter, open label, randomized, controlled phase II trial	90	Apixaban	Apixaban 10 mg orally twice daily for the first 7 days and 5 mg twice daily.	6 months	Efficacy outcome: - recurrent VTE Safety outcome: - Major bleeding - Clinically relevant bleeding	64 (39–77) *	56.8%	86.4%	NR	NR
			Dalteparin	Subcutaneous dalteparin (200 IU/kg for 1 month followed by 150 IU/kg once daily).			63 (42–78) *	50.0%	71.7%	NR	NR
McBane et al., 2020 [31]	Randomized and open trial	287	Apixaban	Apixaban 10 mg orally twice daily for the first 7 days and 5 mg twice daily.	6 months	Efficacy outcome: - recurrent VTE Safety outcome: - Major bleeding - No major clinically relevant bleeding - Major bleeding plus clinically relevant non-major bleeding	64.4	48.0%	65.3%	9.3%	6.7%
			Dalteparin	Subcutaneous dalteparin (200 IU/kg for 1 month followed by 150 IU/kg once daily).			64.0	48.7%	66.0%	9.3%	8.7%

Abbreviations: PE, pulmonary embolism; VTE: venous thromboembolism; NR: Not reported; * Median (range).

**Table 2:** Summary of GRADE results.

Outcome	Anticipated Absolute Effects * (95% CI)		Relative Effect (95% CI)	N°. of Participants (Studies)	Certainty of the Evidence (GRADE)
	Risk with Dalteparin	Risk with Apixaban			
Efficacy: Recurrent venous thromboembolism	73 for 1000	37 for 1000 (12 to 112)	RR 0.50 (0.16 to 1.53)	1501 (3 Randomized Controlled Experiments)	⨁◯◯◯ Very low ^a,b^
Safety: - Major bleeding	35 for 1000	45 for 1000 (11 to 186)	RR 1.29 (0.31 to 5.27)	1501 (3 Randomized Controlled Experiments)	⨁◯◯◯ Very low ^a,b^
- No major clinically relevant bleeding	60 for 1000	91 for 1000 (63 to 131)	RR 1.52 (1.05 to 2.19)	1501 (3 Randomized Controlled Experiments)	⨁◯◯◯ Very low ^a,b^

* The risk in the intervention group (and its 95% confidence interval) is based on the assumed risk in the comparison group and the relative effect of the intervention (and its 95% confidence interval). CI: confidence interval; RR: Risk ratio. “⨁” There is certainty of the evidence. “◯” There is no certainty of the evidence. GRADE Working Group grades of evidence. High certainty: We are very confident that the true effect is close to that of the estimate of effect. Moderate certainty: We are moderately confident in the effect estimate: the true effect is likely to be close to the effect estimate, but there is a possibility that it is substantially different. Low certainty: Our confidence in the effect estimate is limited: the actual effect may be substantially different from the effect estimate. Very low certainty: We have very little confidence in the estimate of effect: The actual effect is likely to be substantially different from the estimate of effect. Explanations: a. The participant knows which drug they are receiving due to its route of administration; therefore, blinding is lost. b. The effects in the evaluated studies are not significant and have a high risk of bias.

## Data Availability

Data are available in the Supporting Information.

## Supplementary Materials

The following supporting information can be downloaded at: https://www.mdpi.com/1648-9144/59/10/1867/s1. Research approach format.
