# Correction: Arce-Huamani et al. Efficacy and Safety of Apixaban versus Dalteparin as a Treatment for Cancer-Associated Venous Thromboembolism: A Systematic Review and Meta-Analysis. *Medicina* 2023, *59*, 1867

**DOI:** 10.3390/medicina60010134

**Published:** 2024-01-11

**Authors:** Miguel A. Arce-Huamani, Joshuan J. Barboza, José Fabián Martínez-Herrera, J. Smith Torres-Roman, Jorge L. Maguiña

**Affiliations:** 1 Facultad de Ciencias de la Salud, Universidad Cientifica del Sur, Lima 15067, Peru; jorge.luis.maguina@gmail.com; 2 Cancer Research Networking, Universidad Cientifica del Sur, Lima 15067, Peru; jfabianmd@gmail.com (J.F.M.-H.); jstorresroman@gmail.com (J.S.T.-R.); 3 Centro de Investigación en Epidemiología y Medicina Basada en Evidencia, Universidad Norbert Wiener, Lima 13007, Peru; jbarbozameca@gmail.com; 4 Cancer Center, Medical Center American British Cowdray, Mexico City 01120, Mexico; 5 Latin American Network for Cancer Research (LAN–CANCER), Lima 11702, Peru

There was an error in the original publication [1]. No statistical significance was observed in clinically relevant major or non-major bleeding, and similarly, in the evaluation of major bleeding and clinically relevant non-major bleeding, there were no statistical differences between the two drugs.

Corrections have been made to the *Abstract and Conclusions*:

Abstract: *Background and Objectives*: Venous thromboembolism (VTE) is common in cancer patients. Anticoagulant therapy with low-molecular-weight heparins (LMWHs) and direct oral anticoagulants (DOACs), such as dalteparin and apixaban, have demonstrated efficacy and safety. However, more comparative research on these drugs is still needed. This study aimed to synthesize evidence on the efficacy of apixaban compared to dalteparin in reducing recurrent VTE, major bleeding, and clinically relevant non-major bleeding associated with cancer. *Materials and Methods*: We systematically searched the PubMed, Scopus, Web of Science, Embase, Cochrane Library, and ClinicalTrials databases up to 5 January 2023 for randomized controlled trials comparing apixaban versus dalteparin as a treatment for cancer-associated VTE. Five studies were included. Effects according to meta-analyses were reported as relative risks (RRs) and their 95% confidence intervals (CIs). *Results*: It was found that 33 of 734 (4.5%) patients treated with apixaban and 56 of 767 (7.3%) with dalteparin had recurrent VTE as an efficacy outcome (RR 0.49, 95% CI 0.15–1.58, I^2^ 38%). Major bleeding occurred in 25 of 734 patients treated with apixaban (3.4%) and 27 of 767 patients treated with dalteparin (3.5%) (RR 1.29, 95% CI 0.31–5.27, I^2^ 59%). Likewise, clinically relevant non-major bleeding occurred in 64 of 734 patients treated with apixaban (8.7%) and 46 of 767 (5.9%) patients treated with dalteparin (RR 1.52, 95% CI 1.05–2.19, I^2^ 0%). *Conclusions*: Apixaban showed a lower risk of recurrent VTE than dalteparin in patients with cancer-associated VTE, albeit with no statistical difference. Statistical significance was observed for no major clinically relevant bleeding but not for major bleeding.

Conclusions: Patients with cancer-associated VTE treated with apixaban showed a lower risk of recurrent VTE (safety outcome) compared to dalteparin, albeit with no statistical difference. In the evaluation of clinically relevant non-major bleeding, there were statistical differences between the two drugs. Likewise, the certainty of the evidence of the studies was very low and the risk of bias was high; thus, it is still not possible to suggest that one of the evaluated treatments is more effective and safer than the other.

This correction was approved by the Academic Editor. The original publication has also been updated.
